# Teaching staff in interprofessional education: A proposed terminology

**DOI:** 10.3205/zma001552

**Published:** 2022-07-15

**Authors:** Gert Ulrich, Hermann Amstad, Olivier Glardon, Sylvia Kaap-Fröhlich

**Affiliations:** 1 Careum Foundation, Department of strategy and innovation, Zurich, Switzerland; 2 amstad-kor, Basel, Switzerland; 3 Society of Swiss Veterinarians, Bern, Switzerland; 4 Zurich University of Applied Sciences, Institute of chemistry and biotechnology, Wädenswil, Switzerland

**Keywords:** terminology, facilitator, supervisor, educator, teacher, interprofessional collaboration

## Abstract

Different terms (e.g., interprofessional, multiprofessional, interdisciplinary) are used in interprofessional education and collaboration without sufficient differentiation or precision in regard to meaning. In recent years academic publications in English and German have contributed to clarifying this issue. However, there are no definitions internationally or in the German-speaking countries (Germany, Austria, Switzerland) specifically referring to the people engaged in teaching interprofessional education. Teaching in interprofessional education has evolved from the traditional role of expert to one of mentor or facilitator. It is also evident that those who teach play a central role in the success of interprofessional courses. While many different designations are used to refer to interprofessional teachers in the relevant literature and in the language of daily use, a uniform and adequate terminology should be used to refer to such teaching staff. Based on literature reviews, this commentary seeks to propose terms for teaching staff active in the area of interprofessional education and thus provide a basis for discussion in the German-speaking countries. Taking the results of the literature analysis and the roles of teachers in interprofessional settings into consideration, we propose that the English term “IP facilitator” (IP for interprofessional) should also be used in the German-speaking world and “facilitateur IP” in the French-speaking world. A French translation is included in attachment 1 to enable broader discussion in Switzerland.

## Abstract (de)

Im Bereich der interprofessionellen Bildung und Zusammenarbeit werden verschiedene Begrifflichkeiten (z.B. interprofessionell, multiprofessionell, interdisziplinär) mitunter wenig trennscharf oder nicht sinngemäß verwendet. In den letzten Jahren haben hier einschlägige Diskussionsbeiträge zur weiteren Klärung in englischsprachigen Publikationen als auch im deutschsprachigen Raum beigetragen. Jedoch finden sich keine internationalen oder gar auf den DACH-Raum (Deutschland, Österreich, Schweiz) bezogenen terminologischen Klärungen zur Benennung der Lehrenden im Bereich der IPE (interprofessional education, IPE). Die Charakteristik von Lehrenden im Bereich der IPE verändert sich von der traditionellen Expert*innenrolle hin zur Rolle von Lernbegleitenden. Zudem ist evident, dass Lehrende eine zentrale Rolle zum Gelingen von interprofessionellen Lehr-/Lernveranstaltungen beitragen. In der einschlägigen Literatur und im alltäglichen Sprachgebrauch finden sich viele verschiedene Bezeichnungen für interprofessionell Lehrende. Daher sollte eine einheitliche und adäquate Benennung dieser Lehrenden erfolgen. Auf Basis von Literaturrecherchen möchte der vorliegende Kommentar Vorschläge zur Bezeichnung von Lehrenden im Feld der IPE und folglich eine Diskussionsgrundlage für den DACH-Raum liefern. Unter Berücksichtigung der vorgenommenen Literaturanalyse und dem damit einhergehenden Verständnis sowie den Rollen von Lehrenden in IPE-Settings, wird die englische Bezeichnung „IP facilitator“ (IP für interprofessionell) auch für den deutschsprachigen Raum bzw. „facilitateur IP“ für den französischsprachigen Raum vorgeschlagen. Im Anhang 1 des Artikels wurde eine französischsprachige Übersetzung des Beitrags angefügt, um so eine breitere Diskussion in der Schweiz zu ermöglichen.

## 1. Introduction

Many terms are used in the context of interprofessional education (IPE) and interprofessional collaborative practice (IPCP) in healthcare, in part without clear differentiation or precision of meaning (inter-, intra-, multi- or transprofessional, inter- or multidisciplinary, etc.). Help in interpreting these terminologies from an international or English-language perspective is found in Khalili et al. [1] and for German speakers in Mitzkat et al. [2]. Those who teach are described as a central factor in the success of interprofessional learning settings [3], [4], [5], [6], [7]. Despite the presence of many confusing terms in the literature to identify and describe this group of people [3], [5], [6], [8], [9], [10], [11], there is, to our knowledge, no clarification of the terminology internationally or in the German-speaking countries (Germany, Austria, Switzerland).

Therefore, the aim of this paper is to propose a uniform terminology based on literature reviews for teaching staff in IPE settings, both in general and specifically for the German-speaking world, along with a French translation for Switzerland. Naturally, this proposal can and should be discussed and debated further.

## 2. Teaching staff in interprofessional education settings

### 2.1. Roles of interprofessional teaching staff

In general, for many teachers engaged in IPE there is a switch from the traditional didactic role as experts to one of interactive, supportive mentors to enable interprofessional education in which the students can learn with, from and about each other [6], [11], [12], [13], [14], [15], [16], [17]. The actual act of educating in IPE is described in the literature with a great degree of consensus as “facilitating” [3], [4], [6], [7]. This specific role should also be captured by an appropriate term for those who teach interprofessionally. Reference is specifically made to Oandasan & Reeves [11], Hylin [15], and Adhikari [12] for detailed explanations of the characteristics, roles and responsibilities of interprofessional teachers.

#### 2.2. Designations for interprofessional teaching staff

Widely varying terms are found in the literature to describe interprofessional teaching staff, such as “facilitator”, “mentor”, “supervisor”, “coach”, “trainer”, “teacher”, “educator”, “counsellor”, “tutor” and even “preceptor”. It is also the case that in different health professions various terms are used for (monoprofessional) teachers or trainers and then applied to IPE in an uncritical manner [8].

The terms “mentor” and “mentoring” are found, for example, in Marshall & Gordon [9], [18] and Arthur & Russell-Mayhew [8]. These authors understand mentoring to mean the creation of conditions under which students and trainees of different professions can learn from and with each other. However, in other publications “mentoring” is described as going beyond educational settings to encompass the personal and professional development of the less experienced person [19], [20]. Explanations of “mentoring” as a concept also describe it as a relationship between only two people (dyadic), which does not explicitly correspond to the interprofessional setting [19].

In contrast to “mentor”, the term “supervisor” is more widely present in the IPE literature, for example, in Oosterom et al. [5] where this term is used to designate those teaching on interprofessional training wards. However, the word “supervisor” is used more traditionally in monoprofessional education [8], [21], [22], in one-on-one situations between “supervisors” and learners, and mainly – like “preceptors” – in practical post-graduate training in healthcare [23], [24]. Hence, the term “supervisor” does not differentiate between monoprofessional and interprofessional education in a way that is distinct in Gribble et al. [22], who refer to profession-specific “supervisors” but to “facilitators” in the interprofessional context.

The term “facilitator” used in reference to interprofessional training staff is found particularly in relevant, often-cited reviews and guidelines [3], [6], [13], [25], [26], [27]. Lie et al. [28], for instance, range from using monoprofessional “clinician educator” to interprofessional “facilitator”, and Brewer & Barr [3] speak not only of the “supervisor” but also the “facilitator”, yet mainly use the term “facilitator” for IPE teachers in their paper. Reeves et al. [6] also use multiple terms (“teacher”, “mentor”, “preceptor” and “supervisor”) while favoring “facilitator” and using it elsewhere. The term “facilitator” is also used outside of clinical IPE, such as in interprofessional classrooms, simulations, and e-learning [14], [16], [29]. Admittedly, the term “facilitator” is not reserved solely for IPE settings. This term is also used in problem-based learning (PBL) [30], where “PBL facilitators” are specifically discussed [30].

Selected German publications speak, in part, of “Lehrende” [31], “Lernbegleitende” [32], [33] and also “facilitators” [33]. When looking at German interprofessional training wards, Mihaljevic et al. [10] and Mette et al. [34] differ by speaking in German of “Lernbegleitende” [10] and “Supervisoren” [34], respectively, while referring to “facilitators” in the English translations of their papers.

Ultimately, the role of interprofessional teaching staff must also be differentiated from that of “educator” or “teacher”. Education refers to two processes that are dependent on each other: teaching and learning, whereby teaching is particularly associated with giving deliberate and planned instruction [12], [35], which contradicts the nature of instruction in IPE settings. Thus, it is impossible to use “educator” or “teacher” as appropriate descriptions in the context of IPE.

#### 2.3. Terms for interprofessional teachers in newer systematic reviews

In addition to this narrative literature review, we wanted to know how frequently different terms were used to describe interprofessional teaching staff in the more recent literature. To answer this question, all systematic reviews from the last seven years containing "interprofessional education" in the title were screened for terminology [6], [29], [36], [37], [38], [39], [40], [41], [42], [43], [44], [45], [46], [47], [48], [49], [50], [51], [52], [53], [54], [55], [56], [57], [58], [59]. Only five of 26 systematic reviews looked at interprofessional teaching staff as a main focus. The term “educator” was used most often, if only peripherally and including the variations “IP educator” and “healthcare educator” (16 reviews). This was followed by “teacher” and “facilitator” (6 reviews each) and “preceptor” (5 reviews). “Supervisor”, “trainer”, “mentor”, or “tutor” were used only sporadically (1-2 reviews). “Coach” and “counsellor” were not found in any of the reviews.

It is evident from this analysis that even in the more recent systematic reviews a variety of terms are still being used for IPE teaching staff without any critical discussion or are simply being borrowed unquestioned from monoprofessional settings and applied to IPE.

## 3. Conclusions for future discussion

Based on our understanding of the roles and responsibilities of IPE teaching staff and taking the literature reviews and analysis into account, we prefer the term “facilitator” as the basis for future discussions. We also recommend this designation be used in the German-speaking world so as to align with the international terminology and avoid causing additional confusion through German translations. For these reasons, we suggest the gender-neutral English word also be used in German. Because the term “facilitator” is also used in other teaching/learning settings (e.g., PBL), “IP” should be placed in front to indicate an interprofessional setting. In conclusion, for IPE teaching staff we propose the terms “IP facilitator” and “facilitateur interprofessionnel/facilitatrice interprofessionnelle” or “facilitateur IP” for the French-speaking regions of Switzerland.

## Note

The French translation of this paper is available as attachment 1 .

## Competing interests

The authors declare that they have no competing interests. 

## Supplementary Material

French version of the paper
