# Supplementary material for: Teaching staff in interprofessional education: A proposed terminology
Source: GMS J Med Educ. 2022 Jul 15;39(3):Doc31. doi: 10.3205/zma001552 (PMC9469565; doi:10.3205/zma001552)
Supplement: French version of the paper [file JME-39-31-s-001.pdf]

French version of the paper

Enseignants de la formation interprofessionnelle: une proposition de terminologie

Gert Ulrich[1], Hermann Amstad[2], Olivier Glardon[3], Sylvia Kaap-Fröhlich[4]

[1] Careum, département Stratégie et innovation, Zurich (CH)

[2] amstad-kor, Bâle (CH)

[3] Société des Vétérinaires Suisses, Berne (CH)

[4] Haute école zurichoise des sciences appliquées, Institut de chimie et de biotechnologie, Wädenswil (CH)

Gert Ulrich, Fondation Careum, gert.ulrich@careum.ch

Hermann Amstad, amstad-kor, contact@amstad-kor.ch

Olivier Glardon, Société des Vétérinaires Suisses, oglardon.president@gstsvs.ch

Sylvia Kaap-Fröhlich, Haute école zurichoise des sciences appliquées, Institut de chimie et de biotechnologie, Wädenswil (CH), kaap@zhaw.ch

Auteur correspondant:

Dr Gert Ulrich

Careum

Département Stratégie et innovation

Pestalozzistrasse 3

CH-8032 Zurich

gert.ulrich@careum.ch

+41 43 222 64 26

Attachment 1 to Ulrich G, Amstad H, Glardon O, Kaap-Fröhlich S. *Teaching staff in interprofessional education: A proposed terminology*. GMS J Med Educ. 2022;39(3):Doc31. DOI: 10.3205/zma001552

Abstract

Dans le domaine de la formation et de la collaboration interprofessionnelles, certains termes (p. ex. interprofessionnel, multiprofessionnel, interdisciplinaire) sont parfois employés de façon imprécise ou inappropriée. Au cours des dernières années, des contributions pertinentes au débat ont participé à une clarification sur ce plan dans les publications tant en anglais qu’en allemand. Toutefois, il n’existe pas de clarifications terminologiques au niveau international ni même au sein de l’espace germanophone (Allemagne, Autriche, Suisse) concernant l’appellation des enseignants intervenant dans le domaine de l’IPE (interprofessional education, formation interprofessionnelle). Les caractéristiques des enseignants IPE évoluent, passant du rôle traditionnel d’expert à celui d’accompagnant de l’apprentissage. De plus, il est évident que les enseignants jouent un rôle essentiel dans la réussite de l’enseignement / l’apprentissage interprofessionnel. Dans la littérature spécialisée et le langage courant, on trouve de nombreuses appellations pour les enseignants interprofessionnels. Il convient donc de définir une appellation uniforme et adéquate. Sur la base de recherches bibliographiques, le présent commentaire soumet des propositions d’appellation des enseignants IPE afin de fournir une base de discussion pour l’espace germanophone. Compte tenu de l’analyse de la littérature réalisée et de la compréhension qui en découle, ainsi que des rôles des enseignants dans les contextes IPE, la dénomination anglaise «IP facilitator» (IP pour interprofessionnel) est également proposée pour l’espace germanophone et «facilitateur IP» pour l’espace francophone. Une traduction en français de l’article est jointe à l’annexe 1 afin de permettre un débat plus large en Suisse.

Mots-clés français: terminologie, intermédiaires, assistants, formateurs, enseignants, collaboration interprofessionnelle

1. Introduction

Dans le contexte de la formation interprofessionnelle (interprofessional education, IPE) et de la collaboration interprofessionnelle (interprofessional collaborative practice, IPCP) dans le système de santé, un grand nombre de termes sont parfois employés de façon imprécise ou inappropriée (inter-, intra-, multi- ou transprofessionnel, inter- ou multidisciplinaire, etc.). Khalili et al [1] pour le contexte international ou anglophone et Mitzkat et al. [2] pour l'espace germanophone fournissent des interprétations terminologiques utiles. Les enseignants sont décrits comme les principaux facteurs de réussite de la formation interprofessionnelle [3-7]. On trouve dans la littérature une diversité terminologique déroutante d'appellations pour les personnes de ce groupe [3], [5], [6], [8-11], mais, à notre connaissance, il n'existe aucune clarification terminologique au niveau international ni même au sein de l'espace germanophone (Allemagne, Autriche, Suisse).

L'objectif de l'article est donc de proposer, sur la base de recherches bibliographiques, une dénomination uniforme des enseignants intervenant dans des contextes IPE de manière générale, mais aussi, plus particulièrement, pour l'espace germanophone et d'en déduire une variante francophone pour la Suisse. Bien entendu, cette proposition peut et doit faire l'objet d'un débat plus approfondi.

2. Enseignants intervenant dans des contextes de formation interprofessionnels

2.1. Rôles des enseignants interprofessionnels

D'une manière générale, l'IPE consiste pour de nombreux enseignants à passer d'un rôle didactique traditionnel d'expert à un rôle interactif de soutien et d'accompagnement visant à permettre aux personnes en formation d'apprendre les uns des autres, avec les autres et à travers les autres [6], [11-17]. Dans la littérature, l'activité d'enseignement proprement dite dans le domaine de l'IPE est assez unanimement définie par le terme «to facilitate» (en français: «faciliter», «permettre», «encourager» ou «soutenir») [3], [4], [6], [7]. Ce rôle spécifique devrait également se refléter dans une appellation appropriée des enseignants interprofessionnels. Pour une description plus détaillée des caractéristiques, rôles et tâches des enseignants interprofessionnels, nous renvoyons notamment aux articles d'Oandasan & Reeves [11], de Hylin [15] et d'Adhikari [12].

2.2. Appellations des enseignants interprofessionnels

La littérature spécialisée utilise divers termes pour désigner les enseignants interprofessionnels, par exemple «facilitator», «mentor», «supervisor», «coach», «trainer», «teacher», «educator», «counsellor», «tutor» ou encore «preceptor». De plus, les appellations des formateurs (monoprofessionnels) peuvent varier selon les professions de santé (p. ex. «mentor» ou «supervisor») et sont transposées de manière irréfléchie au domaine de l'IPE [8].

La notion de «mentoring» ou de «mentor» se retrouve par exemple chez Marshall & Gordon [9], [18] et chez Arthur & Russell-Mayhew [8]. Ces auteurs entendent par «mentorat» la création de conditions permettant aux personnes en formation d'apprendre avec et des étudiants d'autres professions. Toutefois, d'autres passages mentionnent que le «mentoring» va au-delà du cadre de la formation et vise un accompagnement global dans la formulation d'idées et le développement personnel et professionnel de la personne moins expérimentée [19], [20]. Les débats sur le terme «mentoring» décrivent en outre une relation entre deux personnes uniquement (dyadiques), ce qui ne correspond pas non plus explicitement aux contextes interprofessionnels [19].

Contrairement au «mentor», la notion de «supervisor» est plus répandue dans la littérature IPE, par exemple chez Oosterom et al. [5], qui l'utilisent pour désigner les personnes accompagnant l'apprentissage dans les lieux de formation interprofessionnels. Toutefois, le terme «supervisor» est plutôt employé traditionnellement pour la formation monoprofessionnelle [8], [21], [22], ainsi que pour les situations individuelles entre un «supervisor» et une personne en formation et surtout – à l'instar du «preceptor» (en français p. ex.: formateur, maître d'apprentissage, instructeur) – pour la pratique post-graduée des soins [23], [24]. Le terme «supervisor» ne fait donc pas une distinction nette entre formation monoprofessionnelle et formation interprofessionnelle, ce qui est clairement exprimé par exemple par Gribble et al. [22], qui parlent de «supervisors» pour les professions spécifiques, mais de «facilitators» dans le contexte interprofessionnel.

En particulier, le terme «facilitator» désigne les enseignants interprofessionnels dans les revues et directives souvent citées dans ce domaine [3], [6], [13], [25-27]. Lie et al. [28] passent par exemple du «clinician educator» monoprofessionnel au «facilitator» interprofessionnel et Brewer & Barr [3] mentionnent aussi bien le «supervisor» que le «facilitator», mais réservent le plus souvent dans leur article la notion de «facilitator» aux enseignants IPE. Reeves et al. [6] identifient également plusieurs appellations appliquées aux enseignants IPE («teacher», «mentor», «preceptor» et «supervisor»), mais préfèrent et emploient le terme «facilitator» dans leur publication. Le terme «facilitator» est également utilisé en dehors des IPE cliniques, par exemple dans les «classrooms», les «simulations» et les «e-learnings» interprofessionnels [14], [16], [29]. Toutefois, le «facilitator» n'est pas cantonné

aux contextes IPE. Dans le domaine de l'apprentissage basé sur des problèmes (problem-based learning, PBL), on retrouve également cette appellation [30], sachant que l'on parle dans certains cas spécifiquement de «PBL facilitators» [30].

Certaines publications germanophones emploient parfois les termes «enseignants» [31], «accompagnants de l'apprentissage» [32],[33] ou «facilitators» [33]. En ce qui concerne les lieux de formation interprofessionnels allemands, Mihaljevic et al. [10] et Mette et al. [34] utilisent «accompagnants l'apprentissage» [10] et «superviseurs» [34]. Dans la traduction anglaise de leurs publications, c'est de nouveau le terme «facilitators» qui est employé.

Enfin, le rôle des enseignants interprofessionnels doit également être délimité en tant qu'«educators» ou que «teachers». Le terme «education» se réfère à deux processus interdépendants: l'enseignement (teaching) et l'apprentissage (learning), le terme «teaching» étant plus particulièrement associé à des instructions délibérées et planifiées [12], [35], ce qui est en contradiction avec l'enseignement de type IPE. Par conséquent, ni «educator» ni «teacher» ne peuvent être des descriptions appropriées pour les enseignants IPE.

2.3. Appellations des enseignants interprofessionnels dans les revues systématiques récentes

Outre cette recherche narrative de la littérature, nous voulions savoir à quelle fréquence les différentes appellations des enseignants interprofessionnels sont utilisées dans la littérature récente. À cet effet, toutes les revues systématiques des sept dernières années dont le titre contenait «interprofessional education» ont été analysées sous l'angle des appellations [6], [29], [36-59]. Les enseignants interprofessionnels constituent un thème central dans seulement 5 des 26 revues systématiques. Le terme le plus employé, même de façon marginale, est «educator», également dans sa variante «IP educator» ou «healthcare educator» (16 revues), suivi de «teacher», de «facilitator» (6 revues chacun) et de «preceptor» (5 revues). «Supervisor», «trainer», «mentor» ou «tutor» ne sont utilisés qu'occasionnellement (dans 1 à 2 revues). «Coach» et «counsellor» n'ont été trouvés dans aucune des revues.

Il ressort clairement de ces analyses que, même dans les revues systématiques récentes, il existe toujours différentes appellations pour les enseignants IPE et qu'elles sont utilisées sans débat de fond ou transposées sans remise en question dans le domaine des IPE à partir de contextes monoprofessionnels.

3. Conclusions comme base de discussion future

En raison de notre compréhension des rôles et des tâches des enseignants IPE et en tenant compte des recherches et analyses de la littérature, nous privilégions l'appellation «facilitator» comme base de discussion future. Nous recommandons également cette désignation pour l'espace germanophone afin de reprendre la terminologie internationale et de ne pas créer de confusion supplémentaire par des traductions allemandes correspondantes. C'est la raison pour laquelle nous proposons, en allemand également, la prononciation anglaise sans distinction de genre. Le terme étant également utilisé dans d'autres contextes d'enseignement / d'apprentissage (p. ex. PBL), il devrait être précédé de la mention «IP» (pour «interprofessionnel»). En résumé, nous proposons l'appellation «IP facilitator» pour les enseignants IPE et «facilitateur interprofessionnel/facilitatrice interprofessionnelle» ou «facilitateur IP» pour les régions francophones de Suisse.

Littérature spécialisée

1. Khalili A, Thistlethwaite J, El-Awaisi A, Pfeifle A, Gilbert J, Lising D, MacMillan K, Maxwell B, Grymonpre R, Rodrigues F, Snyman S, Xyrichis A. Guidance on Global Interprofessional Education and Collaborative Practice Research: Discussion Paper. InterprofessionalResearch.Global & Interprofessional.Global; 2019. Zugänglich unter/available from: https://interprofessionalresearch.global/global-ipecp-guidance/
2. Mitzkat A, Berger S, Reeves S, Mahler C. More terminological clarity in the interprofessional field - a call for reflection on the use of terminologies, in both practice and research, on a national and international level. GMS J Med Educ. 2016;33(2):Doc36. DOI: 10.3205/zma001035
3. Brewer ML, Barr H. Interprofessional Education and Practice Guide No. 8: Team-based interprofessional practice placements. J Interprof Care. 2016;30(6):747-753. DOI: 10.1080/13561820.2016.1220930
4. Freeth D, Reeves S. Learning to work together: using the presage, process, product (3P) model to highlight decisions and possibilities. J Interprof Care. 2004;18(1):43-56. DOI: 10.1080/13561820310001608221
5. Oosterom N, Floren LC, Ten Cate O, Westerveld HE. A review of interprofessional training wards: Enhancing student learning and patient outcomes. Med Teach. 2019;41(5):547-554. DOI: 10.1080/0142159X.2018.1503410
6. Reeves S, Fletcher S, Barr H, Birch I, Boet S, Davies N, McFadyen A, Rivera J, Kitto S. A BEME systematic review of the effects of interprofessional education: BEME Guide No. 39. Med Teach. 2016;38(7):656-668. DOI: 10.3109/0142159X.2016.1173663
7. Ulrich G, Amstad H, Glardon O, Kaap-Frohlich S. Interprofessional education in the Swiss healthcare system: situation analysis, perspectives and roadmap. Zürich: Careum; 2020. Zugänglich unter/available from: http://www.careum.ch/workingpaper9-long
8. Arthur N, Russell-Mayhew S. Preparing Counsellors for Interprofessional Collaboration through Supervision and Lateral Mentoring. Can J Couns. 2010;44(3):258-271.
9. Marshall M, Gordon F. Interprofessional Mentorship: Taking on the Challenge. J Integr Care. 2005;13(2):38-43. DOI: 10.1108/14769018200500016
10. Mihaljevic AL, Schmidt J, Mitzkat A, Probst P, Kenngott T, Mink J, Fink CA, Ballhausen A, Chen J, Cetin A, Murrmann L, Müller G, Mahler C, Götsch B, Trierweiler-Hauke B. Heidelberger

Interprofessionelle Ausbildungsstation (HIPSTA): a practice- and theory-guided approach to development and implementation of Germany's first interprofessional training ward. GMS J Med Educ. 2018;35(3):Doc33. DOI: 10.3205/zma001179
11. Oandasan I, Reeves S. Key elements for interprofessional education. Part 1: the learner, the educator and the learning context. J Interprof Care. 2005;19 Suppl 1:21-38. DOI: 10.1080/13561820500083550
12. Adhikari S. Facilitation Vs Teaching: 20 Differences We Must Know! Public Health Notes. 2019. Zugänglich unter/available from: https://www.publichealthnotes.com/facilitation-vs-teaching-20-differences-we-must-know/
13. Evans S, Shaw N, Ward C, Hayley A. "Refreshed...reinforced...reflective": A qualitative exploration of interprofessional education facilitators' own interprofessional learning and collaborative practice. J Interprof Care. 2016;30(6):702-709. DOI: 10.1080/13561820.2016.1223025
14. Hayward K, Brown M, Pendergast N, Nicholson M, Newell J, Fancy T, Cameron H. IPE via online education: Pedagogical pathways spanning the distance. J Interprof Educ Pract. 2021;24:100447. DOI: 10.1016/j.xjep.2021.100447
15. Hylin U. Interprofessional education: Aspects on learning together on an interprofessional training ward (PhD). Stockholm: Karolinska Institutet; 2010.
16. Reeves S, Pelone F, Hendry J, Lock N, Marshall J, Pillay L, Wood R. Using a meta-ethnographic approach to explore the nature of facilitation and teaching approaches employed in interprofessional education. Med Teach. 2016;38(12):1221-1228. DOI: 10.1080/0142159X.2016.1210114
17. Sargeant J, Hill T, Breau L. Development and testing of a scale to assess interprofessional education (IPE) facilitation skills. J Contin Educ Health Prof. 2010;30(2):126-131. DOI: 10.1002/chp.20069
18. Marshall M, Gordon F. Exploring the role of the interprofessional mentor. J Interprof Care. 2010;24(4):362-374. DOI: 10.3109/13561820903275001
19. Sambunjak D, Marusic A. Mentoring: what's in a name? JAMA. 2009;302(23):2591-2592. DOI: 10.1001/jama.2009.1858
20. Shah SK, Nodell B, Montano SM, Behrens C, Zunt JR. Clinical research and global health: mentoring the next generation of health care students. Glob Public Health. 2011;6(3):234-246. DOI: 10.1080/17441692.2010.494248
21. Bedford S, Repa L, Renouf A. Supervision in Interprofessional Education: Benefits, Challenges, and Lessons Learned. J Psychother Integr. 2020;30(1):16-24. DOI: 10.1037/int0000167
22. Gribble N, Ladyshewsky RK, Parsons R. Strategies for interprofessional facilitators and clinical supervisors that may enhance the emotional intelligence of therapy students. J Interprof Care. 2017;31(5):593-603. DOI: 10.1080/13561820.2017.1341867
23. Omer TA, Suliman WA, Moola S. Roles and responsibilities of nurse preceptors: Perception of preceptors and preceptees. Nurse Educ Pract. 2016;16(1):54-59. DOI: 10.1016/j.nepr.2015.07.005
24. Snowdon DA, Leggat SG, Taylor NF. Does clinical supervision of healthcare professionals improve effectiveness of care and patient experience? A systematic review. BMC Health Serv Res. 2017;17(1):786. DOI: 10.1186/s12913-017-2739-5
25. Evans S, Ward C, Shaw N, Walker A, Knight T, Sutherland-Smith W. Interprofessional education and practice guide No. 10: Developing, supporting and sustaining a team of facilitators in online interprofessional education. J Interprof Care. 2020;34(1):4-10. DOI: 10.1080/13561820.2019.1632817
26. Godden-Webster A, Murphy G. Interprofessional Collaboration in practice: A guide for strengthening student learning experiences. Halifax, Nova Scotia: Dalhousie University Faculty of Health Professions; 2014.
27. Willgerodt MA, Abu-Rish Blakeney E, Brock DM, Liner D, Murphy N, Zierler B. Interprofessional education and practice guide No. 4: Developing and sustaining

interprofessional education at an academic health center. J Interprof Care. 2015;29(5):421-425. DOI: 10.3109/13561820.2015.1039117
28. Lie DA, Forest CP, Kysh L, Sinclair L. Interprofessional education and practice guide No. 5: Interprofessional teaching for prequalification students in clinical settings. J Interprof Care. 2016;30(3):324-330. DOI: 10.3109/13561820.2016.1141752
29. Evans S, Ward C, Reeves S. Online interprofessional education facilitation: A scoping review. Med Teach. 2019;41(2):215-222. DOI: 10.1080/0142159X.2018.1460656
30. Hmelo-Silver CE, Barrows HS. Goals and strategies of a problem-based learning facilitator. Interdiscip J Probl Based Learn. 2006;1(1):21-39. DOI: 10.7771/1541-5015.1004
31. Walkenhorst U, Mahler C, Aistleithner R, Hahn EG, Kaap-Frohlich S, Karstens S, Reiber K, Stock-Schröer B, Sottas B. Position statement GMA Committee--"Interprofessional Education for the Health Care Professions". GMS Z Med Ausbild. 2015;32(2):Doc22. DOI: 10.3205/zma000964
32. Bode S, Hinrichs J, Ballnus R, Straub C, Mette M. Schulungskonzept für Lernbegleitende auf interprofessionellen Ausbildungsstationen. PADUA. 2021;16(1):45-50. DOI: 10.1024/1861-6186/a000595
33. Sottas B. Handbuch für Lernbegleiter auf interprofessionellen Ausbildungsstationen. Stuttgart: Robert Bosch Stiftung; 2020.
34. Mette M, Baur C, Hinrichs J, Oestreicher-Krebs E, Narciss E. Implementing MIA - Mannheim's interprofessional training ward: first evaluation results. GMS J Med Educ. 2019;36(4):Doc35. DOI: 10.3205/zma001243
35. Bastable SB. Nurse as educator: Principles of teaching and learning for nursing practice. Boston, MA: Jones & Bartlett Learning; 2003.
36. Almoghirah H, Nazar H, Illing J. Assessment tools in pre-licensure interprofessional education: A systematic review, quality appraisal and narrative synthesis. Med Educ. 2021;55(7):795-807. DOI: 10.1111/medu.14453
37. Berger-Estilita J, Fuchs A, Hahn M, Chiang H, Greif R. Attitudes towards Interprofessional education in the medical curriculum: a systematic review of the literature. BMC Med Educ. 2020;20(1):254. DOI: 10.1186/s12909-020-02176-4
38. Do H, Calache H, Darby I, Lau P. The effectiveness of interprofessional education for the management of diabetes and oral health: A systematic review. J Interprof Care. 2021;35(3):454-463. DOI: 10.1080/13561820.2020.1758046
39. Dyess AL, Brown JS, Brown ND, Flautt KM, Barnes LJ. Impact of interprofessional education on students of the health professions: a systematic review. J Educ Eval Health Prof. 2019;16:33. DOI: 10.3352/jeehp.2019.16.33
40. El-Awaisi A, Joseph S, El Hajj MS, Diack L. A comprehensive systematic review of pharmacy perspectives on interprofessional education and collaborative practice. Res Social Adm Pharm. 2018;14(10):863-882. DOI: 10.1016/j.sapharm.2017.11.001
41. Guraya SY, Barr H. The effectiveness of interprofessional education in healthcare: A systematic review and meta-analysis. Kaohsiung J Med Sci. 2018;34(3):160-165. DOI: 10.1016/j.kjms.2017.12.009
42. Herath C, Zhou Y, Gan Y, Nakandawire N, Gong Y, Lu Z. A comparative study of interprofessional education in global health care: A systematic review. Medicine (Baltimore). 2017;96(38):e7336. DOI: 10.1097/MD.0000000000007336
43. Jackson M, Pelone F, Reeves S, Hassenkamp AM, Emery C, Titmarsh K, Greenwood N. Interprofessional education in the care of people diagnosed with dementia and their carers: a systematic review. BMJ Open. 2016;6(8):e010948. DOI: 10.1136/bmjopen-2015-010948
44. Kangas S, Rintala TM, Jaatinen P. An integrative systematic review of interprofessional education on diabetes. J Interprof Care. 2018;32(6):706-718. DOI: 10.1080/13561820.2018.1500453

45. Kent F, Keating JL. Interprofessional education in primary health care for entry level students--A systematic literature review. Nurse Educ Today. 2015;35(12):1221-1231. DOI: 10.1016/j.nedt.2015.05.005
46. Kossioni AE, Marchini L, Childs C. Dental participation in geriatric interprofessional education courses: A systematic review. Eur J Dent Educ. 2018;22(3):e530-e541. DOI: 10.1111/eje.12348
47. Marcussen M, Nørgaard B, Arnfred S. The Effects of Interprofessional Education in Mental Health Practice: Findings from a Systematic Review. Acad Psychiatry. 2019;43(2):200-208. DOI: 10.1007/s40596-018-0951-1
48. O'Carroll V, McSwiggan L, Campbell M. Health and social care professionals' attitudes to interprofessional working and interprofessional education: A literature review. J Interprof Care. 2016;30(1):42-49. DOI: 10.3109/13561820.2015.1051614
49. Riskiyana R, Claramita M, Rahayu GR. Objectively measured interprofessional education outcome and factors that enhance program effectiveness: A systematic review. Nurse Educ Today. 2018;66:73-78. DOI: 10.1016/j.nedt.2018.04.014
50. Rodrigues da Silva Noll Goncalves J, Noll Goncalves R, da Rosa SV, Schaia Rocha Orsi J, Moyses SJ, Iani Werneck R. Impact of interprofessional education on the teaching and learning of higher education students: A systematic review. Nurse Educ Pract. 2021;56:103212. DOI: 10.1016/j.nepr.2021.103212
51. Rutherford-Hemming T, Lioce L. State of Interprofessional Education in Nursing: A Systematic Review. Nurse Educ. 2018;43(1):9-13. DOI: 10.1097/NNE.0000000000000405
52. Shen N, Yufe S, Saadatfard O, Sockalingam S, Wiljer D. Rebooting Kirkpatrick: Integrating Information System Theory Into the Evaluation of Web-based Continuing Professional Development Interventions for Interprofessional Education. J Contin Educ Health Prof. 2017;37(2):137-146. DOI: 10.1097/CEH.0000000000000154
53. Shrader S, Farland MZ, Danielson J, Sicat B, Umland EM. A Systematic Review of Assessment Tools Measuring Interprofessional Education Outcomes Relevant to Pharmacy Education. Am J Pharm Educ. 2017;81(6):119. DOI: 10.5688/ajpe816119
54. Siddiqi A, Zafar S, Sharma A, Quaranta A. Diabetes mellitus and periodontal disease: The call for interprofessional education and interprofessional collaborative care - A systematic review of the literature. J Interprof Care. 2022;36(1):93-101. DOI: 10.1080/13561820.2020.1825354
55. Spaulding EM, Marvel FA, Jacob E, Rahman A, Hansen BR, Hanyok LA, Martin SS, Han HR. Interprofessional education and collaboration among healthcare students and professionals: a systematic review and call for action. J Interprof Care. 2021;35(4):612-621. 10.1080/13561820.2019.1697214
56. Visser CLF, Ket JCF, Croiset G, Kusurkar RA. Perceptions of residents, medical and nursing students about Interprofessional education: a systematic review of the quantitative and qualitative literature. BMC Med Educ. 2017;17(1):77. DOI: 10.1186/s12909-017-0909-0
57. Wang Z, Feng F, Gao S, Yang J. A Systematic Meta-Analysis of the Effect of Interprofessional Education on Health Professions Students' Attitudes. J Dent Educ. 2019;83(12):1361-1369. DOI: 10.21815/JDE.019.147
58. Welsch LA, Hoch J, Poston RD, Parodi VA, Akpinar-Elci M. Interprofessional education involving didactic TeamSTEPPS(R) and interactive healthcare simulation: A systematic review. J Interprof Care. 2018;32(6):657-665. DOI: 10.1080/13561820.2018.1472069
59. Winter IP, Ingledew PA, Golden DW. Interprofessional Education in Radiation Oncology. J Am Coll Radiol. 2019;16(7):964-971. DOI: 10.1016/j.jacr.2018.12.022
